# SELFormerMM: multimodal molecular representation learning via SELFIES, structure, text, and knowledge graph integration

**DOI:** 10.1093/bioinformatics/btag451

**Published:** 2026-08-21

**Authors:** Erva Ulusoy, Şevval Bostancı, Bora Engin Deniz, Tunca Doğan

**Affiliations:** Biological Data Science Lab, Department of Computer Engineering, Hacettepe University, Ankara, 06800, Turkey; Department of Bioinformatics, Graduate School of Health Sciences, Hacettepe University, Ankara, 06100, Turkey; Biological Data Science Lab, Department of Computer Engineering, Hacettepe University, Ankara, 06800, Turkey; Biological Data Science Lab, Department of Computer Engineering, Hacettepe University, Ankara, 06800, Turkey; Biological Data Science Lab, Department of Computer Engineering, Hacettepe University, Ankara, 06800, Turkey; Department of Bioinformatics, Graduate School of Health Sciences, Hacettepe University, Ankara, 06100, Turkey; Department of Health Informatics, Institute of Informatics, Hacettepe University, Ankara, 06800, Turkey

## Abstract

**Motivation:**

Molecular representation learning is central to computational drug discovery. However, most existing models rely on single-modality inputs, such as molecular sequences or graphs, which capture only limited aspects of molecular behaviour. Yet unifying these modalities with complementary resources, such as textual descriptions and biological interaction networks, into a coherent multimodal framework remains non-trivial, hindering the development of more informative and biologically grounded representations.

**Results:**

We introduce SELFormer Multimodal (SELFormerMM), a multimodal molecular representation learning framework that integrates SELFIES notations with structural graphs, textual descriptions and knowledge graph–derived biological interaction data. By aligning these heterogeneous views, SELFormerMM effectively captures complementary signals that unimodal approaches often overlook. Our performance evaluation has revealed that SELFormerMM outperforms structure-, sequence- and knowledge-based models on multiple molecular property prediction tasks. Ablation analyses further indicate that effective cross-modal alignment and modality coverage improve the model’s ability to exploit complementary information. Overall, integrating SELFIES with structural, textual and biological context enables richer molecular representations and provides a promising framework for hypothesis-driven drug discovery.

**Availability and implementation:**

SELFormerMM is available as a programmatic tool at https://github.com/HUBioDataLab/SELFormerMM. Pre-trained and fine-tuned model checkpoints, multimodal pre-training and finetuning datasets including SMILES, textual descriptions and knowledge graph, and their precomputed single-modality embedding files are available at https://huggingface.co/datasets/HUBioDataLab/SELFormerMM.

## 1 Introduction

Accurate molecular representation learning lies at the core of modern computational drug discovery and cheminformatics. The ability to encode chemical compounds into informative vector representations directly impacts a wide range of downstream applications, including molecular property prediction, virtual screening, toxicity estimation, and *de novo* drug design. Over the past decade, advances in deep learning have significantly improved our capacity to model complex structure–property relationships, enabling data-driven approaches that complement and often surpass traditional descriptor-based methods. As molecular datasets continue to grow in scale and diversity, developing robust and transferable representation learning frameworks has become increasingly critical for reliable predictive modelling across heterogeneous biological tasks.

Recent progress in molecular representation learning has largely centred on sequence-based and graph-based approaches. Sequence-based models formulate molecular learning as a language modelling problem by treating chemical strings as textual sequences, and the derivatives of the transformer architecture are frequently employed to learn contextualized molecular representations (Fabian *et al.* 2020, Irwin *et al.* 2022, Ross *et al.* 2022, Singh *et al.* 2026). The most widely used representation, SMILES (Simplified Molecular-Input Line-Entry System), encodes molecular graphs as linear character sequences (Wang *et al.* 2019). However, SMILES has several limitations: the same molecule can be represented by multiple non-canonical strings, syntactically valid strings may correspond to chemically invalid structures and structural constraints are only implicitly captured (Krenn *et al.* 2020). To address these issues, SELFIES (Self-Referencing Embedded Strings) was introduced as a grammar-constrained representation that guarantees 100% validity and improves syntactic robustness (Krenn *et al.* 2020). Transformer-based models trained on SELFIES have demonstrated competitive and often superior performance to SMILES-based counterparts across various molecular property prediction tasks, highlighting the benefits of chemically constrained tokenization for molecular representation learning (Born and Manica 2023, Yüksel *et al.* 2023).

Molecular graph-based methods represent compounds as structured relational graphs in which atoms correspond to nodes and chemical bonds to edges. Graph neural networks operating on these molecular graphs employ message-passing mechanisms to learn embeddings that capture local chemical environments as well as higher-order topological dependencies (Yang *et al.* 2019, Wang *et al.* 2022). Variants incorporating geometric information further enhance the ability to model structure-sensitive properties (Fang *et al.* 2022, Liu *et al.* 2022, Zhou *et al.* 2023).

Molecular representations can also be informed by textual and structured relational knowledge sources. Text-based approaches leverage natural language descriptions, such as curated database entries or automatically generated summaries, to extract semantic embeddings using pre-trained language models (Balaji *et al.* 2023, Liu *et al.* 2023b). Knowledge graphs (KGs) represent biochemical entities, including compounds, proteins, genes, and diseases, together with their multi-relational interactions, enabling embeddings informed by relational domain knowledge (Doǧan *et al.* 2021, Xie *et al.* 2024, Şen *et al.* 2026).

Although these approaches have substantially advanced molecular modelling, unimodal methods typically learn representations confined to a single molecular perspective. Sequence, structure, text, and knowledge-based encodings capture complementary aspects of molecular identity, yet they are often developed and optimized independently. Molecules are inherently multimodal entities: they possess syntactic encodings, topological structure, semantic annotations, and rich biological interaction contexts. This observation has motivated the development of multimodal learning frameworks that integrate heterogeneous molecular modalities into a unified representation space (Liu *et al.* 2023a, 2024, Zhang *et al.* 2024). Nevertheless, most current approaches focus on a limited subset of modalities and primarily align views in pairwise or partially integrated settings. The incorporation of large-scale biological interaction networks and the learning of a shared embedding space that simultaneously harmonizes structural, semantic, and relational information remain as promising directions for advancing multimodal molecular representation learning.

In this work, we propose SELFormer Multimodal (SELFormerMM), a unified multimodal molecular representation learning framework that integrates SELFIES encodings, 2D molecular graphs, natural-language molecular descriptions, and biological interaction embeddings obtained from a comprehensive KG within a shared latent space. Building upon a transformer-based SELFIES encoder (Yüksel *et al.* 2023), SELFormerMM incorporates graph, text and KG modalities through dedicated projection networks and aligns heterogeneous molecular views using a multi-view contrastive objective under a self-supervised learning paradigm. The model is pre-trained on a large-scale multimodal dataset comprising ∼3 million molecules, with the aim of learning concise, flexible, and semantically meaningful molecular representations that generalize across diverse downstream tasks in drug discovery and development. We evaluate the quality and transferability of the learned embeddings by fine-tuning the model on multiple molecular property prediction benchmarks. By enabling scalable and holistic molecular representations, SELFormerMM provides a practical foundation for advancing generalizable molecular modelling strategies in computational chemistry and drug discovery.

## 2 Materials and methods

SELFormerMM learns shared molecular embeddings by jointly leveraging sequence-based representations, structural graph information, natural language descriptions, and their multi-relational biological interactions modelled in a KG format.

**Figure 1:**
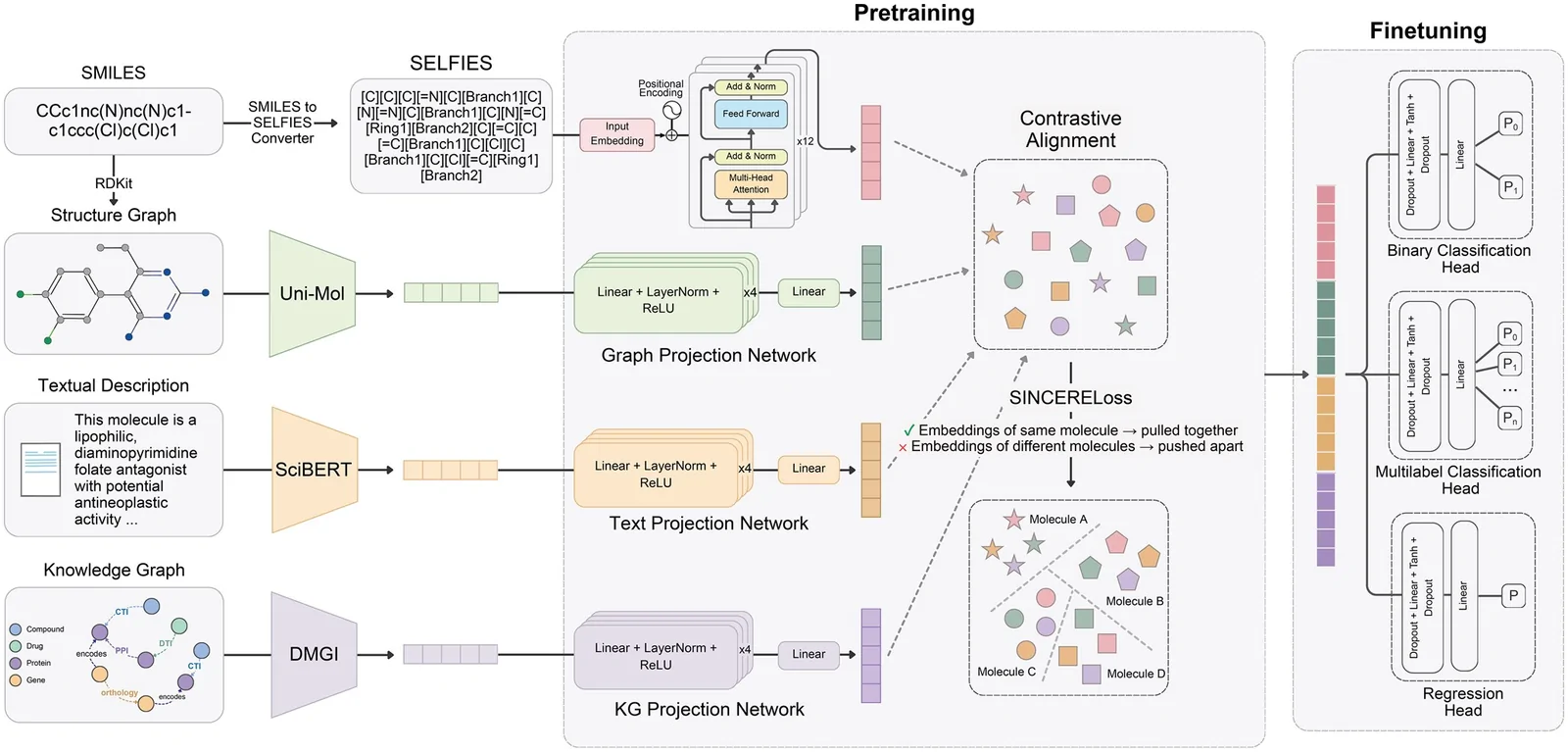
Overview of the SELFormerMM framework. (A) Contrastive multimodal pre-training integrates molecular information from four complementary modalities: SELFIES sequences, structural graphs, textual descriptions and knowledge graph interactions, processed by modality-specific encoders and projection networks, then aligned in a shared representation space via contrastive learning with the SINCERE loss. (B) Downstream task fine-tuning adapts the pre-trained multimodal backbone for molecular property prediction, using task-specific prediction heads for binary classification, multilabel classification and regression.

SELFormerMM is trained in two stages: (i) multimodal pre-training using supervised contrastive learning and (ii) supervised fine-tuning for downstream molecular property prediction tasks. In the pre-training phase, the model aligns modality-specific representations of the same molecule within a shared latent space, enforcing cross-modal consistency (Fig. 1A). In the fine-tuning phase, the pre-trained multimodal backbone is adapted to task-specific objectives by attaching a classification or regression head (Fig. 1B).

### 2.1 Multimodal pre-training

#### 2.1.1 Dataset

To construct the multimodal pre-training dataset, we aggregated molecular data from complementary large-scale resources, ensuring coverage of sequence, text, KG and structure modalities.

For the molecular sequence modality, we used the ChEMBL v36 (Mendez *et al.* 2019) database, from which molecular SMILES representations for 2 854 815 molecules were obtained. To incorporate textual descriptions, we utilized the M3-20M dataset (Guo *et al.* 2024), a large-scale multimodal molecular dataset containing natural language descriptions for over 20 million molecules, derived from multiple sources including PubChem annotations, physicochemical property-based descriptions, and GPT-3.5-generated summaries. Since the molecules in M3-20M are indexed independent of ChEMBL, we constructed a mapping procedure to associate ChEMBL molecules with their corresponding textual descriptions. Mapping was first performed using standard InChIKeys, which provide a canonical chemical identifier independent of string representations. For molecules that could not be matched using InChIKeys, we applied exact SMILES matching. Remaining unmatched molecules were further processed by canonicalizing SMILES strings in both datasets before attempting an additional mapping step. Through this multi-stage mapping procedure, textual descriptions were successfully assigned to 492 702 molecules.

To incorporate structured biological interaction networks of molecules, we utilized the CROssBARv2-KG (https://crossbarv2.hubiodatalab.com/, https://github.com/HUBioDataLab/CROssBARv2) (Şen *et al.* 2026), which integrates large-scale biological data from 34 heterogeneous sources. To reduce memory consumption and mitigate noise during embedding generation, we constructed a focused subgraph containing only the node and edge types most directly associated with molecular properties. Specifically, we retained the node types Compound, Protein, Drug, and Gene, along with the biologically meaningful relationships connecting them: compound–target interaction (CTI), drug–target interaction (DTI), protein–protein interaction (PPI), and orthology relationships. The resulting graph contains 726 254 compound nodes, 14 594 drug nodes, 199 479 gene nodes, and 461 775 protein nodes, totalling 1 402 102 nodes. It includes 1 353 738 CTI edges, 60 500 DTI edges, 64 856 orthology edges, and 2 945 736 PPI edges, amounting to 4 424 830 relationships in total. Since CROssBARv2 represents compounds using ChEMBL identifiers, molecules in the ChEMBL v36 dataset could be directly associated with KG nodes. Through this mapping, 725 908 molecules were linked to their corresponding KG nodes.

For the structural graph modality, molecular graphs were derived directly from SMILES strings. Prior to graph construction, SMILES were validated using RDKit (Landrum 2013), and molecules for which RDKit failed to generate a valid molecule were discarded. Finally, to prepare the sequence modality for model input, all SMILES strings in the dataset were converted into SELFIES representations using the SELFIES API (Krenn *et al.* 2020). The distribution of modality availability across the final pre-training dataset is summarized in Table 1.

**Table 1:** Number of available molecules per modality in the final pre-training dataset.^a^

Modality	No. of molecules
SMILES	2 854 815
Text description	492 702
Knowledge graph	725 908
Structural graph	2 854 734^a^

a Eighty-one molecules that failed RDKit validation were excluded from the structural-graph modality.

#### 2.1.2 Model architecture and implementation

SELFormerMM comprises four modality-specific branches and a shared projection space that aligns representations from different modalities (Fig. 1A).

For the sequence modality, we employed SELFormer (Yüksel *et al.* 2023), a RoBERTa-based chemical language model that tokenizes SELFIES sequences using bracket-delimited, grammar-aware segmentation. The molecule-level representation is the contextualized embedding of the [CLS] token. During multimodal pre-training, the SELFormer backbone and projection networks are jointly trained from scratch, enabling adaptation to the multimodal alignment objective.

For the text modality, we employed SciBERT (Beltagy *et al.* 2019), a BERT-based model pre-trained on scientific text, to encode M3-20M molecular descriptions. Molecule-level 768-dimensional embeddings were obtained via mean pooling over token representations.

For the structural graph modality, we utilized Uni-Mol (Zhou *et al.* 2023), a universal 3D molecular pre-training framework trained on 209 million 3D molecular conformations. Uni-Mol generates molecule-level embeddings that capture atomic connectivity and 3-dimensional structural information derived from SMILES representations. We used Uni-Mol’s molecule-level representation (cls_repr) output as the fixed-dimensional structural embedding, resulting in 512-dimensional graph representations.

To encode biological interaction information, we employed the DMGI (Deep Mutual Graph Infomax) architecture (Park *et al.* 2020). DMGI models heterogeneous graphs using relation-specific graph convolution layers and is trained with a mutual information maximization objective that contrasts true node features with corrupted counterparts. The model was trained on the CROssBARv2-KG subgraph, with compound node features initialized using pre-trained SELFormer embeddings. After training, relation-specific compound representations were aggregated via mean pooling to obtain a single 128-dimensional KG embedding per compound.

The text, structural graph, and KG encoder models were initialized from pre-trained checkpoints and kept frozen during multimodal training. We defined a non-linear projection network for each of these modalities to map their native embedding spaces into the 768-dimensional hidden space of the SELFormer encoder (Fig. 1A). Each projection head is implemented as a multilayer perceptron with intermediate dimensional expansion layers, followed by LayerNorm and ReLU activations, and a final linear transformation that projects to the shared hidden dimension *H*. If a modality is unavailable, a zero vector is passed through the corresponding projection network, enabling the model to learn dedicated missing-modality representations.

To align modality-specific representations within a unified embedding space, we optimize SELFormerMM using a multi-view, multi-positive supervised contrastive objective. The objective encourages embeddings derived from different modalities of the same molecule to become similar, while pushing apart embeddings belonging to different molecules. We implement this objective using SINCERELoss, a supervised extension of the InfoNCE formulation that accommodates multiple positive views per instance (Equation 1).


(1) $$L=-\frac{1}{\left|I\right|}\sum_{i\in I}\frac{1}{\left|P(i)\right|}\sum_{j\in P(i)}\log\frac{\exp\left(S_{\mathrm{ij}}/\tau \right)}{\sum_{k\in A(i)}\exp\left(S_{\mathrm{ik}}/\tau \right)}$$

*E_i_* denotes the embedding vector of the *i*-th view in the batch (e.g. sequence, graph, text, or KG representation of a molecule). *S_ij_* is the cosine similarity between embeddings *E_i_* and *E_j_*. τ is a temperature parameter set to 0.07. *I* denotes the set of all anchor indices in the batch. For a given anchor *i*, *P(i)* is the set of positive indices corresponding to embeddings of the same molecule across different modalities, excluding the trivial self-pair. *A(i)* denotes all embeddings in the batch except the anchor itself, comprising both its positives and negatives, where negatives correspond to embeddings of different molecules.

The model was implemented in PyTorch using the Hugging Face Transformers library. SELFormerMM comprises 246 245 376 trainable parameters. Multimodal pre-training used a random 90/10 train–validation split and was optimized with the AdamW optimizer on four NVIDIA RTX A5000 GPUs. The model was trained for 25 epochs using mixed-precision optimization, requiring ∼3 days to complete.

### 2.2 Fine-tuning

To evaluate the quality and transferability of the learned multimodal representations, we fine-tuned SELFormerMM on nine MoleculeNet benchmarks (Wu *et al.* 2018) (Fig. 1B). The selected datasets span both classification and regression tasks and cover a wide range of dataset sizes and biological endpoints. For binary classification, we used BBBP (blood–brain barrier penetration), HIV (inhibition of HIV replication), and BACE (binding to human β-secretase 1). Multilabel classification was evaluated on Tox21 (toxicity prediction) and SIDER (side effect prediction). For regression, we included ESOL (aqueous solubility), FreeSolv (hydration free energy), Lipophilicity, and PDBbind (protein–ligand binding affinity prediction). Detailed dataset statistics and modality availability for each task are provided in Table S1, available as supplementary data at *Bioinformatics* online.

For downstream adaptation, we attach a task-specific prediction head to the pre-trained SELFormerMM backbone. The backbone produces four modality-specific embeddings per molecule: the sequence representation from the RoBERTa [CLS] token and the projected embeddings of the structural graph, text and KG modalities. These four vectors are concatenated along the feature dimension to form a single multimodal representation, which serves as input to the prediction head.

Since MoleculeNet datasets provide SMILES strings as the only molecular identifier, modality matching was performed via SMILES using the same procedure applied to the M3-20M dataset: standard InChIKey matching followed by exact and canonicalized SMILES matching, which nevertheless resulted in missing modalities for many samples (Table S1, available as supplementary data at *Bioinformatics* online).

Missing modalities were represented using zero vectors prior to concatenation. The prediction module maps the multimodal embedding to task-specific outputs: either class probabilities for classification tasks or continuous values for regression tasks. To balance stability and adaptability, we adopt a partial-freezing strategy: the embedding layer and the first nine transformer blocks of the RoBERTa backbone are kept fixed, while the final three blocks, the modality projection MLPs, and the downstream head are fine-tuned. This preserves the pre-trained multimodal alignment while enabling task-specific refinement at higher layers.

We follow the recommended MoleculeNet protocols for evaluation. Regression and multilabel datasets are randomly split, whereas scaffold splitting is applied to the binary classification tasks using the Chemprop implementation (Heid *et al.* 2024). Each dataset is divided into 80/10/10 train, validation, and test splits. Hyperparameters, including backbone and head learning rates, weight decay, batch size, and number of epochs, are tuned separately for each dataset using the validation split, while final performance is reported on the held-out test split.

For classification tasks, we use cross-entropy loss (single-label) and binary cross-entropy per label (multilabel) and evaluate performance using the area under the ROC curve (ROC-AUC). For regression tasks, we optimize mean squared error loss (MSE) and report root mean square error (RMSE). Formal definitions of the evaluation metrics and loss functions are provided in Section S1, available as supplementary data at *Bioinformatics* online.

## 3 Results

### 3.1 Pre-training and optimization

We examined how multimodal pre-training shapes the representation space on the validation split by analysing intra- and inter-molecule cosine similarities and computing their separation margin. During training, intra-molecule similarity increased substantially from 0.016 to 0.522, while inter-molecule similarity rose more moderately from 0.229 to 0.378. As a result, the separation margin improved from −0.206 to 0.144, indicating that representations of the same molecule across different modalities became more strongly aligned while preserving separation between representations of different molecules.

Local neighbourhood structure was further assessed using 5-nearest-neighbour (kNN) accuracy for molecule-identity classification in the embedding space. Validation kNN accuracy improved from 0.20 to 0.62, reflecting increasingly coherent and label-consistent clustering behaviour. Collectively, these results demonstrate that contrastive pre-training produced structured and discriminative multimodal molecular embeddings suitable for downstream adaptation.

### 3.2 Molecular property prediction benchmarks

We evaluated the effectiveness of the learned representations by fine-tuning SELFormerMM on downstream molecular property prediction tasks. We adopt the standard MoleculeNet evaluation setup (80/10/10: train/validation/test), selecting models based on validation dataset performance and reporting final results on the held-out test set.

We apply scaffold splits to binary classification benchmarks and random splits to regression and multilabel classification benchmarks, as recommended by the MoleculeNet benchmark suite (Wu *et al.* 2018). Results are reported as mean ± standard deviation across three independent random seeds. All baseline results in Tables 2 and 3 are taken from the original publications under the same conventions. However, as the baselines were trained under heterogeneous experimental conditions (e.g. different random seeds and optimization procedures), marginal performance differences should be interpreted with caution. Baseline models for which results under the corresponding split type were not available are excluded from the respective tables. Scaffold-based splitting constitutes a more stringent out-of-distribution assessment and often yield lower absolute performance.

**Table 2:** Performance comparison on classification-based molecular property prediction benchmarks.^a^

Model type	Model name	Input type	SIDER	BACE	BBBP	HIV	Tox21
Unimodal	MolBERT	SMILES	–	0.866 ± 0.000	0.762 ± 0.000	0.783 ± 0.000	–
	ChemBERTa		–	0.781 ± 0.019	0.700 ± 0.027	0.740 ± 0.013	–
	CLM		0.659 ± 0.040	0.861 ± 0.040	**0.915 ± 0.020**	**0.813 ± 0.030**	**0.858 ± 0.050**
	D-MPNN	2D structure	0.676 ± 0.014	–	0.738 ± 0.001	0.776 ± 0.008	0.851 ± 0.002
	MolCLR		–	**0.890 ± 0.003**	0.738 ± 0.002	0.806 ± 0.011	–
	MGCN		–	0.734 ± 0.030	0.850 ± 0.064	0.738 ± 0.016	–
	GraphMVP-C	3D structure	–	0.812 ± 0.009	0.724 ± 0.016	0.770 ± 0.012	–
	GEM		–	0.856 ± 0.011	0.724 ± 0.004	0.806 ± 0.009	–
	Uni-Mol		–	0.857 ± 0.002	0.729 ± 0.006	0.808 ± 0.003	–
	MolXPT	Text	–	0.884 ± 0.010	0.800 ± 0.005	0.781 ± 0.004	–
	GPT-MolBERTa		–	0.734 ± 0.005	0.741 ± 0.002	0.755 ± 0.013	–
	ReaKE	KG	–	0.781	–	0.751	0.824
	SELFormer (ours)	SELFIES	**0.724 ± 0.007**	0.822 ± 0.019	0.891 ± 0.046	0.632 ± 0.042	0.658 ± 0.006
Multimodal	FP-GNN	2D structure, fingerprint	0.661	0.860	0.916	**0.824**	0.815
	MoleculeSTM	2D structure, text	–	0.808 ± 0.013	0.700 ± 0.005	0.769 ± 0.018	–
	MolLM	2D structure, 3D structure, text	–	0.841 ± 0.009	0.757 ± 0.017	0.802 ± 0.007	–
	MvMRL	SMILES, 2D structure, fingerprint	0.653 ± 0.027	**0.878 ± 0.336**	**0.963 ± 0.019**	–	**0.845 ± 0.011**
	GIT-Mol	SMILES, 2D structure, text	–	0.811 ± 0.015	0.739 ± 0.006	–	–
	SELFormerMM (ours)	SELFIES, text, 2D structure and KG	**0.751 ± 0.013**	0.779 ± 0.021	0.947 ± 0.005	0.788 ± 0.025	**0.845 ± 0.012**

a Results are reported as the area under the ROC-AUC metric (higher is better). Scaffold splits are used for BACE, BBBP, and HIV, while random splits are used for Tox21 and SIDER, as recommended by the benchmark suite (Wu *et al.* 2018). SELFormer and SELFormerMM results are reported as mean ± standard deviation across three independent random seeds. Baseline model results are taken from original publications. Within each model category (unimodal and multimodal), the best-performing results are highlighted in bold, and the second-best results are underlined. “–” indicates that results were not reported for the corresponding method–task combination. Results for MGCN are taken from Wang *et al.* (2022).

**Table 3:** Performance comparison on regression-based molecular property prediction benchmarks.^a^

Model type	Model name	Input type	ESOL	FreeSolv	Lipophilicity	PDBbind
Unimodal	MolBERT	SMILES	**0.531 ± 0.040**	**0.948 ± 0.330**	0.561 ± 0.030	–
	ChemFormer		0.633 ± 0.040	1.230 ± 0.300	0.598 ± 0.020	–
	D-MPNN	2D structure	0.555 ± 0.047	1.075 ± 0.054	**0.555 ± 0.023**	1.391 ± 0.012
	SS-GNN	Protein-ligand complex	–	–	–	**1.181 ± 0.047**
	OnionNet		–	–	–	1.278
	RT	SELFIES	0.690 ± 0.010	1.030 ± 0.250	0.740 ± 0.020	–
	SELFormer (ours)		0.601 ± 0.074	1.104 ± 0.399	0.792 ± 0.026	1.373 ± 0.015
Multimodal	FP-GNN	2D structure, fingerprint	0.675	0.905	0.625	**1.296**
	MvMRL	SMILES, 2D structure and fingerprint	**0.601 ± 0.055**	**0.832 ± 0.128**	0.634 ± 0.018	–
	SELFormerMM (ours)	SELFIES, text, 2D structure and KG	0.672 ± 0.060	1.070 ± 0.065	**0.624 ± 0.049**	1.310 ± 0.040

a Results are reported as root mean square error (RMSE) (lower is better). Random splits are used for all datasets, as recommended by the benchmark suite (Wu *et al.* 2018). SELFormer and SELFormerMM results are reported as mean ± standard deviation across three independent random seeds. Baseline results are taken from original publications. Within each model category (unimodal and multimodal), the best-performing results are highlighted in bold, and the second-best results are indicated with underlining. “–” indicates that results were not reported for the corresponding method–task combination.

#### 3.2.1 Classification tasks

Among the unimodal models evaluated on classification tasks (Table 2), SELFormer achieves the highest ROC-AUC on SIDER (0.724) and performs competitively on BBBP (0.891), closely approaching the top-performing CLM (0.915) (Born *et al.* 2023). This performance is likely supported by the SELFIES representation, which enforces structural validity and simplifies stereochemistry, providing a robust foundation for predicting complex pharmacological responses (Yüksel *et al.* 2023). CLM achieves the strongest unimodal results on BBBP (0.915) and also performs strongly on HIV (0.813) and Tox21 (0.858), likely benefiting from SMILES augmentation strategies that improve representation robustness, particularly on larger datasets. On BACE, structure-based MolCLR (0.890) (Wang *et al.* 2022) and the text-based MolXPT (0.884) (Liu *et al.* 2023b) achieve the strongest unimodal results, reflecting the advantage of their large-scale pre-training on tens of millions of molecules.

Integrating additional modalities substantially improves SELFormer across most classification benchmarks. SELFormerMM achieves the best performance on SIDER (0.751), suggesting that multimodal alignment enables the model to capture complementary biological and chemical information relevant to adverse-effect prediction. On BBBP, SELFormerMM achieves 0.947 ROC-AUC, ranking second overall behind MvMRL (0.963) (Zhang *et al.* 2024), whose integration of SMILES, molecular graphs, and fingerprint descriptors appears particularly effective for permeability-related tasks.

SELFormerMM also improves markedly over SELFormer on HIV, increasing the ROC-AUC from 0.632 to 0.788. A similar trend is observed on Tox21, where SELFormerMM achieves 0.845 ROC-AUC, matching the best multimodal result reported by MvMRL while substantially outperforming SELFormer (0.658). Together, these results indicate that cross-modal alignment provides complementary predictive signals across both binary and multilabel molecular property prediction tasks, particularly when functional and biological context is important for the target property.

The only classification benchmark on which SELFormerMM underperforms SELFormer is BACE (0.779 versus 0.822), where textual descriptions are available for only 7% of molecules, substantially below the average coverage across the remaining benchmarks (∼65%). Under these conditions, the contrastive alignment objective frequently encounters zero-vector projections for missing modalities, potentially weakening the contribution of multimodal alignment during training.

Models combining 2D molecular structures with fingerprint descriptors, such as FP-GNN (Cai *et al.* 2022) and MvMRL, consistently perform strongly across benchmarks. Fingerprints encode predefined substructural and physicochemical patterns that complement graph-based connectivity information, while both modalities can be directly derived from SMILES representations, ensuring broad modality coverage. This combination likely contributes to their stable and competitive performance across diverse classification tasks.

#### 3.2.2 Regression tasks

Among the unimodal models evaluated on regression tasks (Table 3), SELFormer exhibits moderate yet generally competitive performance across benchmarks. On ESOL, it achieves an RMSE of 0.601, remaining close to the leading unimodal models MolBERT (0.531) (Fabian *et al.* 2020) and D-MPNN (0.555) (Yang *et al.* 2019). On FreeSolv and Lipophilicity, SELFormer performs comparably to RT (Born and Manica 2023), another model based on SELFIES representation. In contrast, on PDBbind, SELFormer (1.373) is surpassed by protein–ligand interaction models: SS-GNN (1.181) (Zhang *et al.* 2022) and OnionNet (1.278) (Zheng *et al.* 2019), which are trained directly on three-dimensional binding complex structures and explicitly capture intermolecular geometric interactions that sequence-based molecular representations cannot readily model. We note that SELFormer results are reported as mean ± standard deviation across three random seeds using hyperparameters optimized under a single-seed procedure in the original study. Since these hyperparameters were not re-tuned separately for each seed, the reported mean scores may underestimate the best achievable performance under seed-specific optimization, which likely contributes to the lower average scores observed relative to the original single-run SELFormer results on some benchmarks (Yüksel *et al.* 2023).

SELFormerMM improves SELFormer’s results on PDBbind, achieving an RMSE of 1.310. This improvement may stem from integrating structural and KG-derived information, which can provide additional biological context relevant to protein–ligand interaction prediction.

On Lipophilicity, SELFormerMM achieves the strongest performance among multimodal models (0.624) and approaches the best unimodal methods. On ESOL, however, SELFormerMM (0.672) underperforms both SELFormer (0.601) and MvMRL (0.601), while slightly outperforming FP-GNN (0.675).

Similarly, on FreeSolv, SELFormerMM achieves an RMSE of 1.070, remaining behind the top-performing MvMRL (0.832) and FP-GNN (0.905). These results suggest that multimodal alignment may be less beneficial for smaller regression datasets dominated by strong physicochemical signals. As also observed in our ablation studies (Tables S2–S5, available as supplementary data at *Bioinformatics* online), incorporating additional modalities such as text or KG representations may introduce optimization noise or gradient interference, potentially weakening the strong structural signal exploited by the unimodal SELFormer model for solubility-related prediction tasks.

These findings are further supported by our benchmark-specific keyword filtering analyses, in which sentences explicitly mentioning the target property were removed from molecular textual descriptions using a curated keyword list (Table S8 and Section S4, available as supplementary data at *Bioinformatics* online). Across the two representative benchmarks evaluated, filtering did not consistently degrade performance, and in one case improved it, suggesting that property-related language in the descriptions introduces semantic noise rather than providing useful predictive information.

### 3.3 Ablation study

To quantify the contribution of individual modalities and training stages, we conducted ablation experiments using identical hyperparameter configurations across all variants to ensure fair comparison. Performance was evaluated on randomly split classification and regression benchmarks.

The SELFIES-based encoder (SELFormer) served as the baseline, with additional modalities (Text, KG, Graph) incorporated sequentially via their respective projection networks. All model variants were pre-trained for 20 epochs to control computational cost, yielding a set of pre-trained checkpoints (Tables S2 and S3 for classification and regression, respectively, under Section S2, available as supplementary data at *Bioinformatics* online).

The impact of downstream fine-tuning was evaluated by taking the 20-epoch pre-trained SELFormerMM checkpoint and fine-tuning it for 50 epochs on each benchmark (Tables S4 and S5, available as supplementary data at *Bioinformatics* online). Pre-trained performance, by contrast, was established using a single-epoch one-pass protocol. Finally, we conducted leave-one-modality-out ablations to evaluate robustness under incomplete modality availability. Starting from the same 20-epoch pre-trained SELFormerMM checkpoint, selected modalities were masked by zeroing their embeddings during fine-tuning, and the resulting models were evaluated alongside the SELFormerMM fine-tuned variant (Tables S4 and S5, available as supplementary data at *Bioinformatics* online).

Across both classification (Table S2, available as supplementary data at *Bioinformatics* online) and regression (Table S3, available as supplementary data at *Bioinformatics* online) tasks, integrating additional modalities generally enhanced performance relative to the SELFormer baseline. An exception was observed for SIDER, where SELFormer already provided a strong baseline (ROC-AUC = 0.724) and the addition of single modalities did not yield substantial improvements. However, the fully multimodal configuration achieved the best overall performance on this dataset (ROC-AUC = 0.729), suggesting that integrated cross-modal representations can offer complementary benefits in certain settings. In contrast, for several other datasets, adding a single complementary modality outperformed the fully multimodal configuration. This likely reflects increased optimization difficulty during contrastive pre-training, where aligning multiple heterogeneous views may introduce gradient interference or cross-modal noise. Among individual modalities, 2D graph information emerged as the most robust signal, yielding the most consistent improvements and highlighting the importance of structural connectivity for molecular property prediction. This was particularly pronounced on the PDBbind dataset, where the SELFormer+Graph pre-trained model (RMSE = 1.333) outperformed even the fully multimodal fine-tuned variants, consistent with the strong dependence of binding affinity prediction on structural interaction patterns.

Fine-tuning generally improved performance across most datasets, confirming that the pre-trained multimodal backbone provides a strong initialization (Tables S4 and S5, available as supplementary data at *Bioinformatics* online). In contrast, performance on the Tox21 dataset remained close to random for both pre-trained (ROC-AUC = 0.500) and fine-tuned variants (0.463). However, task-specific hyperparameter optimization improves performance on this dataset (0.845; Table 2), indicating that the framework can learn meaningful signals when appropriately tuned.

Leave-one-modality-out experiments further highlight the importance of structural information (Tables S4 and S5, available as supplementary data at *Bioinformatics* online). Removing the graph modality typically degraded performance, whereas removing text or KG modalities produced less consistent effects. This behaviour likely reflects differences in modality coverage: graph representations are available for nearly all molecules, while textual and KG information are available only for a subset. When a modality is available for only part of the dataset, the resulting mixture of informative and zero-valued embeddings can introduce optimization instability, bias the model towards uneven feature utilization, and create feature inconsistency across samples (Reza *et al.* 2025). Overall, multimodal gains depend not only on modality richness but also on coverage consistency and dataset-specific characteristics. A coverage-balanced subset analysis on Tox21 and Lipophilicity, which have the highest numbers of fully covered molecules (Table S1, available as supplementary data at *Bioinformatics* online), further confirms that auxiliary modalities can provide stronger signals when modality coverage is balanced, while also indicating that their contribution remains dataset- and modality-dependent. Detailed results are provided in Supplementary Data (Section S3 and Tables S6 and S7, available as supplementary data at *Bioinformatics* online).

### 3.4 Use-case analysis

We evaluated the biochemical relevance of our molecular property predictions through case studies using representative molecules from three example downstream tasks: BBBP, SIDER, and FreeSolv.

The BBBP task is a binary classification problem that predicts whether a compound can permeate the blood–brain barrier (BBB), a highly selective barrier that regulates the entry of molecules into the central nervous system (CNS). BBB permeability is therefore a key determinant in the development of CNS-targeting drugs. For this task, we selected two molecules indicated for neurological disorders: dextroamphetamine and benserazide. SELFormerMM predicts dextroamphetamine as BBB-permeable with a probability of 0.998. This prediction is consistent with its known pharmacological activity as a CNS stimulant used to treat narcolepsy and attention deficit hyperactivity disorder (https://go.drugbank.com/drugs/DB01576), reflecting its established ability to cross the BBB (Berman *et al.* 2009). In contrast, benserazide is predicted to be BBB-impermeable (0.006). Clinically, benserazide is administered with levodopa in the treatment of Parkinson’s disease and acts as a peripheral decarboxylase inhibitor that does not cross the BBB, preventing peripheral dopamine formation while allowing levodopa to reach the brain (Roche Products Limited 2020).

Case studies on the SIDER and FreeSolv benchmarks are presented in Section S5, available as supplementary data at *Bioinformatics* online.

## 4 Discussion

In this study, we introduced SELFormerMM, a multimodal molecular representation learning framework that integrates SELFIES, structural graphs, textual descriptions and KG-derived interaction data within a shared latent space via contrastive pre-training on ∼3 million molecules.

Evaluation on MoleculeNet benchmarks demonstrates strong performance on adverse-effect prediction, blood–brain barrier penetration, and lipophilicity, indicating that multimodal alignment enables the model to capture complementary chemical and biological signals. Ablation experiments revealed that the contribution of each modality depends strongly on its characteristics and coverage within the dataset. While structural graph information emerges as the most stable and consistently informative modality, text and KG modalities exhibit more variable effects, which can largely be attributed to their limited coverage across molecules.

Unlike prior multimodal models that either rely on fingerprints or 2D molecular graphs with near-complete coverage, or achieve high modality coverage by training on smaller curated datasets (e.g. 200–300k molecules), SELFormerMM operates at a substantially larger scale. Despite the inherent sparsity of textual and relational data, integrating these heterogeneous modalities still provides complementary signals and improves prediction performance. While datasets with complete modality coverage would enable a more seamless comparison of multimodal architectures, we use the widely adopted MoleculeNet benchmarks to ensure comparability with established baselines.

Case studies further validate that the representations learned by SELFormerMM capture biologically meaningful signals and produce predictions consistent with known pharmacological knowledge.

Several directions remain open for future work. Handling missing modalities more robustly, for instance, via dynamic contrastive objectives that exclude unavailable views, could improve alignment stability. In parallel, our ongoing work explores fully trainable modality-specific encoders instead of frozen ones, potentially improving multimodal learning at the cost of increased computational complexity. To contextualize the current computational footprint of SELFormerMM, runtime and parameter comparisons against representative baselines are provided in Section S6 (Table S9, available as supplementary data at *Bioinformatics* online). Beyond architectural improvements, improving the quality of modality-specific data may also enhance multimodal learning. Training on datasets where textual descriptions are curated from the literature may provide both better coverage and higher information quality. Extending the framework to additional downstream tasks, such as drug–target interaction prediction and *de novo* molecular design, represents a promising direction for demonstrating its utility in real-world drug discovery pipelines. Finally, developing a user-friendly graphical interface will be essential to making SELFormerMM accessible to experimental researchers in biology, chemistry, and drug discovery. Additional discussion on the limitations and future extensions of SELFormerMM is provided in Section S7, available as supplementary data at *Bioinformatics* online.

Overall, SELFormerMM demonstrates that integrating complementary chemical and biological signals yields richer molecular representations, enabling more effective exploration of chemical space and prioritization of biologically relevant candidates for drug discovery. We shared SELFormerMM as a programmatic tool, together with its datasets and pre-trained models, at https://github.com/HUBioDataLab/SELFormerMM.

## Supplementary Material

btag451_Supplementary_Data
