# Supplementary material for: SELFormerMM: multimodal molecular representation learning via SELFIES, structure, text, and knowledge graph integration
Source: Bioinformatics. 2026 Aug 21;42(Suppl 2):btag451. doi: 10.1093/bioinformatics/btag451 (PMC13501325; doi:10.1093/bioinformatics/btag451)
Supplement: Supplementary file 1 [file btag451_supplementary_data.pdf]

Supplementary Data

SELFormerMM: multimodal molecular representation learning via SELFIES, structure, text, and knowledge graph integration

Erva Ulusoy[1,2], Şevval Bostancı[1], Bora Engin Deniz[1], and Tunca Doğan[1,2,3,*]

[1]Biological Data Science Lab, Dept. of Computer Engineering, Hacettepe University, Ankara, Turkey

[2]Dept. of Bioinformatics, Graduate School of Health Sciences, Hacettepe University, Ankara, Turkey

[3]Dept. of Health Informatics, Institute of Informatics, Hacettepe University, Ankara, Turkey

*To whom correspondence should be addressed: Tunca Doğan (tuncadogan@gmail.com)

S1. MoleculeNet dataset statistics and evaluation metrics

Table S1. Overview of the molecular property prediction tasks used for model finetuning, along with dataset statistics, modality coverage, and evaluation protocols.

Dataset	Type of the task	# of compounds	SMILES	Text description	Knowledge graph	Structural graph	# of fully covered compounds	Split type	Metric
BBBP	Binary classification	2039	2039	1376	55	2039	38	Scaffold	ROC-AUC
HIV	Binary classification	41127	41127	5852	320	41120	115	Scaffold	ROC-AUC
BACE	Binary classification	1513	1513	112	198	1513	32	Scaffold	ROC-AUC
SIDER	Multilabel classification	1427	1427	1151	27	1299	20	Random	ROC-AUC
Tox21	Multilabel classification	7831	7831	6011	361	7823	294	Random	ROC-AUC
ESOL	Regression	1128	1128	996	40	1128	39	Random	RMSE
FreeSolv	Regression	642	642	588	31	642	30	Random	RMSE
Lipophilicity	Regression	4200	4200	1352	1010	4200	220	Random	RMSE
PDBbind	Regression	9880	9880	2338	490	9871	145	Random	RMSE

For classification tasks, we use cross-entropy (Eq. SE1) for single-label and binary cross-entropy per label for multilabel prediction.

$$\mathcal{L}_{\mathrm{CE}} = -\frac{1}{N}\sum_{i=1}^{N}\sum_{c=1}^{C} y_{ic} \log p_{ic} \tag{SE1}$$

where N is the number of observations, C is the number of target classes, yic is the ground-truth indicator for sample i and class c, and pic is the predicted softmax probability for class c.

Model performance on classification tasks is evaluated using the Area Under the Receiver Operating Characteristic Curve (ROC-AUC). The ROC curve plots the True Positive Rate (TPR) (Eq. SE2) against the False Positive Rate (FPR) (Eq. SE3) across decision thresholds.

$$\mathrm{TPR} = \frac{TP}{TP+FN} \tag{SE2}$$

$$\mathrm{FPR} = \frac{FP}{FP+TN} \tag{SE3}$$

where TP, TN, FP, and FN denote true positives, true negatives, false positives, and false negatives, respectively.

For regression tasks, we optimise the Mean Squared Error (MSE) loss and report Root Mean Square Error (RMSE) for evaluation (Eq. SE4).

$$\mathrm{RMSE} = \sqrt{\mathrm{MSE}} = \sqrt{\frac{1}{N}\sum_{i=1}^{N}(y_i - p_i)^2} \tag{SE4}$$

S2. Ablation study results

Table S2. Ablation study results on classification-based molecular property prediction benchmarks for pretrained model variants. Results are reported as the area under the receiver operating characteristic curve (ROC-AUC) (higher is better) using random splits for all datasets. All variants were pretrained for 20 epochs; performance was established using a single-epoch one-pass protocol. SELFormer denotes the SELFIES-based baseline encoder. SELFormer+[Modality] indicates pretrained variants with a single additional modality integrated via its modality-specific projection network, and SELFormerMM represents the pretrained model with all modalities included. Best results are shown in bold.

Model name	SIDER	BACE	BBBP	HIV	Tox21
SELFormer	0.724	0.507	0.615	0.548	0.500
SELFormer+Text	0.722	**0.750**	0.724	**0.647**	0.500
SELFormer+KG	0.722	0.718	0.727	**0.647**	0.500
SELFormer+Graph	0.724	0.745	**0.748**	0.640	0.500
SELFormerMM	**0.729**	0.609	0.694	0.581	**0.500**

Table S3. Ablation study results on regression-based molecular property prediction benchmarks for pretrained model variants. Results are reported as root mean square error (RMSE) (lower is better) using random splits for all datasets. All variants were pretrained for 20 epochs; performance was established using a single-epoch one-pass protocol. SELFormer denotes the SELFIES-based baseline encoder. SELFormer+[Modality] indicates pretrained variants with a single additional modality integrated via its modality-specific projection network, and SELFormerMM represents the pretrained model with all modalities included. Best results are shown in bold.

Model name	ESOL	FreeSolv	Lipophilicity	PDBbind
SELFormer	2.812	5.304	1.208	1.664
SELFormer+Text	1.927	4.132	0.938	1.353
SELFormer+KG	1.880	4.801	0.991	1.347
SELFormer+Graph	**1.460**	3.705	**0.928**	**1.333**
SELFormerMM	1.468	**3.656**	1.015	1.368

Table S4. Ablation study results on classification-based molecular property prediction benchmarks for fine-tuned model variants. Results are reported as area under the receiver operating characteristic curve (ROC-AUC) (higher is better) using random splits for all datasets. All variants were fine-tuned for 50 epochs from the 20-epoch pretrained SELFormerMM checkpoint. SELFormerMM denotes the fine-tuned model with all modalities included, and SELFormerMM-No [Modality] denotes leave-one-modality-out settings in which the corresponding modality embeddings were masked by zeroing during finetuning. Best results are shown in bold.

Model name	SIDER	BACE	BBBP	HIV	Tox21
SELFormerMM	**0.730**	0.782	0.825	0.710	**0.463**
SELFormerMM-No text	0.696	0.793	**0.845**	**0.711**	0.463
SELFormerMM-No KG	0.696	**0.802**	0.801	0.707	0.463
SELFormerMM-No graph	0.701	0.787	0.818	0.701	0.463

Table S5. Ablation study results on regression-based molecular property prediction benchmarks for fine-tuned model variants. Results are reported as root mean square error (RMSE) (lower is better) using random splits for all datasets. All variants were fine-tuned for 50 epochs from the 20-epoch pretrained SELFormerMM checkpoint. SELFormerMM denotes the fine-tuned model with all modalities included, and SELFormerMM-No [Modality] denotes leave-one-modality-out settings in which the corresponding modality embeddings were masked by zeroing during finetuning. Best results are shown in bold.

Model name	ESOL	FreeSolv	Lipophilicity	PDBbind
SELFormerMM	0.675	**0.927**	0.600	**1.341**
SELFormerMM-No text	**0.617**	1.050	**0.594**	1.343
SELFormerMM-No KG	0.672	0.959	0.603	1.350
SELFormerMM-No graph	0.716	1.124	0.601	1.363

S3. Coverage-balanced subset analysis

To disentangle the contribution of the multimodal architecture from the effects of modality-coverage sparsity, we repeated the ablation experiments on coverage-balanced subsets of two downstream benchmarks. For each task, we restricted the dataset to molecules for which all four modalities were simultaneously available (Table S1). We selected one classification and one regression benchmark with the largest number of fully covered molecules: Tox21 (n = 294) and Lipophilicity (n = 220), respectively. The same ablation variants, hyperparameter configurations, and 80/10/10 random split protocol used in Tables S2–S5 were applied to these subsets. Results are reported in Tables S6–S7.

On Tox21, all pretrained variants evaluated on the full dataset yield near-random performance (ROC-AUC ≈ 0.500; Table S2). On the coverage-balanced subset, however, the pretrained variants exhibit markedly different behaviour. In particular, the text and KG modalities improve performance relative to pretrained SELFormer (0.479), with SELFormer+Text and SELFormer+KG reaching ROC-AUC values of 0.621 and 0.603, respectively. Pretrained SELFormerMM also surpasses the pretrained SELFormer baseline (0.500 vs. 0.479), indicating that auxiliary modalities become more informative when modality availability is controlled. In contrast, incorporating the graph modality decreases performance (0.460), suggesting that structural connectivity alone may be insufficient to capture the heterogeneous toxicity patterns represented in Tox21, particularly under the substantially smaller fully covered training setting.

On Lipophilicity, the coverage-balanced subset analysis again reveals more pronounced modality-specific effects than those observed on the full benchmark. Relative to pretrained SELFormer (RMSE = 2.292), pretrained SELFormerMM improves performance substantially (1.147), while incorporating text and graph modalities individually also yields strong improvements, with SELFormer+Text and SELFormer+Graph reaching RMSE values of 1.116 and 1.159, respectively. In contrast, adding the KG modality alone decreases performance (2.321), suggesting that relational information may be more difficult to optimise effectively in small fully covered training settings. This trend is also reflected in the finetuning experiments, where the best result is achieved by SELFormerMM-No KG (0.589), while all leave-one-modality-out configurations outperform the fully multimodal SELFormerMM model (0.959).

Unlike Tox21, where all pretrained variants on the original dataset remained close to random performance, the Lipophilicity benchmark already exhibits meaningful signal learning under the standard benchmark setting. Consequently, the fully covered subset appears to expose optimisation trade-offs between modalities more directly, particularly for the KG branch, whose relational information may be less aligned with physicochemical property prediction than textual or structural representations. In addition, the substantially smaller fully covered subset may further amplify optimisation instability and increase sensitivity to modality-specific noise during multimodal training.

Table S6. Coverage-balanced subset ablation results for pretrained model variants. Results are reported as ROC-AUC (higher is better) for Tox21 and RMSE (lower is better) for Lipophilicity, using random splits. Each benchmark was restricted to molecules for which all four modalities are simultaneously available (Tox21: n=294, Lipophilicity: n=220). All variants were pretrained for 20 epochs; performance was established using a single-epoch one-pass protocol. SELFormer denotes the SELFIES-based baseline encoder. SELFormer+[Modality] indicates pretrained variants with a single additional modality integrated via its modality-specific projection network, and SELFormerMM represents the pretrained variant incorporating all four modalities. Best results are shown in bold.

Model name	Tox21	Lipophilicity
SELFormer	0.479	2.292
SELFormer+Text	**0.621**	**1.116**
SELFormer+KG	0.603	2.321
SELFormer+Graph	0.460	1.159
SELFormerMM	0.500	1.147

Table S7. Coverage-balanced subset ablation results for fine-tuned model variants. Results are reported as ROC-AUC (higher is better) for Tox21 and RMSE (lower is better) for Lipophilicity, using random splits. Each benchmark was restricted to molecules for which all four modalities are simultaneously available (Tox21: n=294, Lipophilicity: n=220). All variants were fine-tuned for 50 epochs from the 20-epoch pretrained SELFormerMM checkpoint. SELFormerMM denotes the fine-tuned variant incorporating all four modalities, and SELFormerMM-No [Modality] denotes leave-one-modality-out settings in which the corresponding modality embeddings were masked by zeroing during finetuning. Best results are shown in bold.

Model name	Tox21	Lipophilicity
SELFormerMM	**0.513**	0.959
SELFormerMM-No text	0.500	0.894
SELFormerMM-No KG	0.500	**0.589**
SELFormerMM-No graph	0.500	0.918

S4. Data leakage analysis on textual descriptions

To assess whether the substantial performance gains associated with the text modality are partly attributable to property-relevant language in the textual descriptions, we conducted a sentence-level keyword filtering analysis across all MoleculeNet benchmarks. We retrained and evaluated SELFormerMM under the same experimental configuration used to produce the main results (Tables 2–3), after removing benchmark-related keyword-containing sentences from affected textual descriptions.

For each benchmark, we compiled a curated list of keywords representing the most common surface forms through which the target property could appear in free text. These included direct property-related terms (e.g., “soluble”, “logP”, “HIV”, “BACE”), closely related synonyms, and qualitative descriptors likely to co-occur with the benchmark labels. For each dataset, we then computed the number and fraction of molecules whose descriptions contained at least one keyword occurrence, which is provided in Table S8. The benchmarks most affected by keyword co-occurrence were the solubility-related regression tasks and Tox21. FreeSolv and ESOL showed the highest proportions of affected molecules, driven primarily by the term “soluble”, while Lipophilicity was similarly affected through “logP”. In Tox21, toxicity-related terms collectively affected a small but non-negligible fraction of molecules. By contrast, BBBP, BACE, HIV, PDBbind, and SIDER exhibited minimal to zero keyword prevalence. Importantly, keyword occurrence alone does not imply label leakage, since many terms appear in broad pharmacological or chemical contexts unrelated to the benchmark labels.

To directly evaluate the impact of property-related language on model performance, we selected Tox21 and FreeSolv as representative test cases: the classification benchmark with the highest absolute number of affected molecules and the regression benchmark with the highest proportion of affected molecules, respectively. We applied sentence-level filtering by removing individual sentences containing any benchmark-specific keywords while preserving the remaining description content.

The filtered model achieves ROC-AUC = 0.811 on Tox21 (versus 0.845 ± 0.012 under standard finetuning; Table 2) and RMSE = 0.700 on FreeSolv (versus 1.070 ± 0.065; Table 3). For FreeSolv, filtering improves performance (lower RMSE), consistent with the removal of imprecise solubility-related language that conflicts with the regression objective. For Tox21, however, filtering modestly reduces performance relative to the updated multi-seed baseline (0.811 vs. 0.845), suggesting that any benefit from removing property-related noise is outweighed by the loss of general chemical context in the descriptions. Taken together, these results indicate that property-related language in textual descriptions does not function as a direct label shortcut: the unfiltered performance on Tox21 reflects task-specific hyperparameter optimisation and general chemical context rather than leakage, and the improvement on FreeSolv after filtering reflects the removal of imprecise qualitative descriptors that conflict with the continuous hydration free-energy regression target. Overall, direct property leakage through textual descriptions is unlikely to be a primary driver of SELFormerMM's MoleculeNet performance. One broader caveat is that property-related language in general-purpose compound descriptions is often semantically imprecise relative to the specific benchmark objectives. For Tox21, generic toxicity statements are only weakly aligned with the 12 distinct endpoint labels and may introduce classification noise. Similarly, for FreeSolv, qualitative descriptors such as "highly soluble" or "poorly soluble" do not encode precise hydration free-energy values and may conflict with the regression objective.

Removing such sentences alters the semantic composition of the descriptions and changes model performance in directions consistent with the removal of imprecise or misaligned content rather than informative label-correlated features. Overall, the near-zero keyword prevalence observed for BBBP, BACE, HIV, and PDBbind, together with the absence of performance gains attributable to keyword presence on the two most affected benchmarks, indicates that SELFormerMM's MoleculeNet performance is unlikely to be explained by direct property leakage through textual descriptions.

Table S8. Per-benchmark keyword search results for the data leakage analysis. For each benchmark, all keywords used in the search are listed along with the number of keyword occurrences across all descriptions, the number of affected molecules (i.e., molecules whose description contains the keyword at least once), the total number of molecules in the benchmark, and the proportion of affected molecules (keywords yielding zero occurrences are omitted from the table). BACE is omitted from the table, as no keyword was detected in any description.

Dataset	Keyword	# of occurrences	Affected # of molecules	Total # of molecules	Affected (%)
FreeSolv	soluble	171	145	642	22.6
FreeSolv	solubility	1	1	642	0.2
FreeSolv	hydration	2	1	642	0.2
ESOL	soluble	194	165	1,128	14.6
ESOL	solubility	4	4	1,128	0.4
ESOL	hydration	2	1	1,128	0.1
Lipophilicity	logP	610	610	4,200	14.5
Tox21	toxic	443	348	7,831	4.4
Tox21	toxicity	48	39	7,831	0.5
Tox21	adverse	22	21	7,831	0.3
Tox21	side effect	14	13	7,831	0.2
SIDER	toxic	43	36	1,427	2.5
SIDER	toxicity	15	12	1,427	0.8
SIDER	side effect	10	9	1,427	0.6
SIDER	adverse	6	4	1,427	0.3
PDBbind	Ki	287	136	9,880	1.4
PDBbind	IC50	7	7	9,880	0.1
PDBbind	Kd	2	2	9,880	0.0
HIV	HIV	25	10	41,127	0.0
HIV	antiviral	11	9	41,127	0.0
HIV	reverse transcriptase	14	6	41,127	0.0
HIV	anti-HIV	3	3	41,127	0.0
BBBP	permeability	1	1	2,039	0.0

S5. Use-case analysis: SIDER and FreeSolv benchmarks

SIDER is a multi-label classification task that groups drug side effects into 27 classes based on the affected system organ. To evaluate whether SELFormerMM captures clinically meaningful toxicity patterns, we analysed the predictions for ibandronate, a bisphosphonate commonly used to treat osteoporosis in postmenopausal women (https://go.drugbank.com/drugs/DB00710). SELFormerMM assigns high prediction scores to several clinically relevant system organ classes, including gastrointestinal disorders (0.99), musculoskeletal and connective tissue disorders (0.81), and infections and infestations (0.71). According to the SIDER database, ibandronate is associated with adverse effects such as back pain, arthralgia, dyspepsia, abdominal pain, nausea, diarrhea, and influenza-like symptoms, reported with frequencies of approximately 2–14% (http://sideeffects.embl.de/drugs/60852). These side effects map directly to the organ-system classes predicted by SELFormerMM, indicating that the model captures clinically meaningful toxicity signals. Importantly, unrelated categories such as “pregnancy, puerperium and perinatal conditions” receive low prediction scores (0.05), further indicating that the model learns biologically plausible toxicity patterns.

FreeSolv is a regression benchmark containing experimentally measured hydration free energies for small organic molecules. To assess whether SELFormerMM captures chemically meaningful solvation patterns, we analysed the agreement between predicted and experimental values across the dataset. SELFormerMM achieves a mean absolute deviation of approximately 0.21 kcal/mol, indicating close agreement with experimental measurements. For instance, the polar amine-containing molecule CCNCC (CHEMBL1189) exhibits strong hydration (experimental −4.07 kcal/mol), which the model accurately predicts (−4.07). In contrast, the hydrophobic alkyl bromide CCCCCCCCBr (CHEMBL156047) shows an unfavourable hydration free energy (0.52 kcal/mol), again closely matched by the prediction (0.53). However, fewer than 1% of the molecules in the dataset (6 out of 642) show an opposite sign between predicted

and experimental hydration free energies. Most of these cases involve small halogenated compounds, such as CF and CC(F)F, which are predicted to have positive hydration free energies while experimentally exhibiting slightly negative values. Such cases likely reflect the subtle balance between hydrophobic substituents and polarization effects in aqueous environments, which can be challenging to capture consistently.

S6. Runtime and computational complexity

To contextualise the computational cost of SELFormerMM, we benchmarked finetuning wall-clock time and inference throughput at a fixed batch size of 64 on two representative tasks: BBBP (binary classification) and Lipophilicity (regression). Comparisons were performed against representative open-source baselines using a single NVIDIA RTX A5000 GPU. Specifically, we selected MolBERT (Fabian *et al.* 2020), a SMILES-based BERT model with a parameter count comparable to SELFormer, and MolLM (Tang *et al.* 2024), the closest publicly available multimodal model in terms of trainable footprint.

Finetuning SELFormerMM requires approximately 18 seconds per epoch on BBBP and 38 seconds on Lipophilicity, compared with roughly 9 and 19 seconds for SELFormer and 7 and 11 seconds for MolBERT, respectively. This increase primarily reflects the additional cost of propagating gradients through the modality-specific projection networks. Compared with MolLM, SELFormerMM exhibits a higher finetuning cost despite similar inference throughput. This difference is partly attributable to the substantially larger parameter count of SELFormerMM, driven mainly by the projection layers, which account for approximately 160M parameters. In contrast, although MolLM is also transformer-based, it employs a substantially smaller feed-forward dimension together with a considerably smaller vocabulary embedding table, resulting in a more compact overall architecture. Nevertheless, the absolute differences in finetuning duration across all models remain within tens of seconds per epoch, making them practically negligible and confirming that all models operate within reasonable computational budgets.

Inference throughput remains broadly comparable across models. SELFormerMM processes approximately 186–267 molecules per second, depending on the task, compared with 212–295 for MolBERT, 235–292 for MolLM, and 252–279 for SELFormer. These results suggest that, despite its higher finetuning cost, the multimodal architecture introduces only a modest inference overhead relative to unimodal baselines. Detailed runtime and parameter comparisons are provided in Supplementary Table S9.

Table S9. Runtime and trainable parameter comparison of SELFormerMM and representative competitive methods.

Model	Input type	BBBP finetuning (s/epoch)	BBBP inference (mol/s)	Lipophilicity finetuning (s/epoch)	Lipophilicity inference (mol/s)	Finetuning trainable parameters
SELFormer	SELFIES	9.24	252	19.04	279	87M
MolBERT	SMILES	7.67	212	11.36	295	86M
SELFormerMM	SELFIES, text, 2D structure, KG	18.30	186	37.70	267	190M
MolLM	2D structure, 3D structure, text	6.93	235	10.02	292	50M

S7. Extended Discussion

Several of the multimodal baselines considered in this study (e.g., MvMRL and FP-GNN) employ fingerprint descriptors derived deterministically from SMILES and therefore achieve near-complete modality coverage across benchmarks. In contrast, SELFormerMM integrates curated textual descriptions and biological knowledge-graph relationships, both of which are intrinsically sparse. As a result, direct comparisons between SELFormerMM and fingerprint-based multimodal frameworks inevitably conflate architectural effects with differences in modality availability. To partially disentangle these

factors, we performed coverage-balanced subset analyses, restricting evaluation to molecules with complete modality availability across all four branches. These experiments indicate that multimodal learning becomes more beneficial when coverage imbalance is removed, while also revealing dataset-dependent instability for certain modalities, particularly the KG branch. In these analyses, SELFormer, which relies solely on SELFIES representations derived from SMILES, serves as an indirect approximation of the performance achievable from fully covered sequence-based modalities, analogous to fingerprint-based approaches. Nevertheless, a fully controlled cross-baseline evaluation under matched modality-coverage constraints remains an important direction for future work.

The occasional underperformance of SELFormerMM relative to the unimodal SELFormer baseline likely reflects the interaction between modality sparsity and multimodal training dynamics rather than an inherent limitation of multimodal integration itself. Because textual and KG modalities are available only for subsets of molecules during pretraining, the model is trained on a heterogeneous mixture of fully informative and partially missing samples, which can bias learning toward the always-available SELFIES branch and reduce the effective contribution of sparse auxiliary modalities. In parallel, aligning multiple heterogeneous modalities within a shared latent space introduces competing learning signals during contrastive pretraining, particularly when auxiliary modalities are sparse or only weakly aligned with the downstream objective. The additional projection networks and cross-modal alignment objectives further increase training complexity, especially in smaller data settings. Together, these factors likely contribute to the optimisation instability observed in some downstream tasks.

The KG branch also reflects an important practical trade-off in large-scale multimodal learning. The CROssBARv2 subgraph employed in this work contains approximately 1.4M nodes and 4.4M edges, making end-to-end backpropagation through DMGI during every contrastive pretraining step computationally infeasible on our hardware. Pretraining the KG encoder offline therefore enables scalable integration of relational information into the multimodal framework, but also limits the extent to which KG representations can adapt jointly with the other modalities during downstream optimisation. At the same time, this separation may improve training stability in settings where the knowledge graph is large, relatively static, and shared across many molecules. Future work exploring jointly trainable KG encoders may therefore enable deeper cross-modal integration and more adaptive relational representations.

The textual modality similarly introduces potential limitations in data quality. The M3-20M corpus combines descriptions from PubChem annotations, physicochemical-property-derived templates, and GPT-3.5-generated summaries, the latter of which may contain factual inaccuracies or semantically imprecise statements. Improving the quality and consistency of textual descriptions, particularly through curation from peer-reviewed biomedical literature, therefore represents another important direction for future multimodal molecular representation learning.

Supplementary References

Fabian B, Edlich T, Gaspar H *et al.* Molecular representation learning with language models and domain-relevant auxiliary tasks. *arXiv* 2020, DOI: 10.48550/arXiv.2011.13230.

Tang X, Tran A, Tan J *et al.* MolLM: a unified language model for integrating biomedical text with 2D and 3D molecular representations. *Bioinformatics* 2024;**40**:i357–68.
